# Diagnostic utility of p501s (prostein) in comparison to prostate specific antigen (PSA) for the detection of metastatic prostatic adenocarcinoma

**DOI:** 10.1186/1746-1596-2-41

**Published:** 2007-10-27

**Authors:** Ming Yin, Rajiv Dhir, Anil V Parwani

**Affiliations:** 1 Department of Pathology, University of Pittsburgh Medical Center, Pittsburgh, Pennsylvania, USA

## Abstract

**Background:**

Immunohistochemical detection of prostate specific antigen (PSA) is widely used to identify metastatic prostatic adenocarcinoma. However, PSA may not be expressed in some poorly differentiated prostatic carcinomas and its immunoreactivity has been found in some non-prostatic tissues. P501s (prostein) is a prostate-specific marker that is expressed in the cytoplasm of benign and malignant prostatic glandular cells. It has not been detected in any other normal or malignant tissues. The purpose of this study was to evaluate the expression of P501s in metastatic prostatic adenocarcinoma and compare its expression with PSA.

**Methods:**

Immunohistochemical stains with anti-P501s antibodies were performed on 5-micron sections of tissue microarray (TMA) specimens. The TMA is constructed with normal donor prostates (NDP), prostatic adenocarcinoma (PRCA), non-neoplastic prostatic tissues adjacent to malignant glands (NAT), benign prostatic hyperplasia (BPH), high-grade prostatic neoplasia (PIN), metastatic adenocarcinoma to lymph nodes (MLN), metastatic adenocarcinoma to other sites (MC), and samples of benign testis, colon, adrenal and kidney. The two groups of metastatic lesions were also subjected to stains with antibodies to PSA. A composite score (ranging from 0 to 3) was assigned to score intensity of staining.

**Results:**

Granular staining pattern of p501s was seen in all benign glands (score = 1.77 – 2.1) and malignant acini (score = 1.52) at the apical aspect of cytoplasm, predominantly adjacent to the nuclei. No staining was observed in controls including testis, colon, adrenal and kidney. The MLN group received a score of 1.0, with 10% of cases negative for p501s. The MC cases had a score of 0.64, with 16.7% of case showing loss of p501s expression. Although the metastatic lesions demonstrated similar rate of negative expression with PSA antibody, only 2 MC cases (3.3%) showed simultaneous negative stains for both P501S and PSA.

**Conclusion:**

P501s is an organ specific marker for benign and malignant prostatic epithelial cells. Its characteristic cytoplasmic stain pattern provides an additional valuable immunomarker for detection of metastatic prostatic malignancy, even though the intensity of its expression is reduced, as in the case with PSA. Simultaneous stains with P501S and PSA will greatly improve the detection rate and identify a significant majority of the metastases.

## Background

Prostatic adenocarcinoma is the most prevalent form of cancer in men and the second leading cause of cancer death in the United States [1,2]. The patient's death is often due to local or distal lymph node involvement and distant metastasis [3]. The metastasis can be the first presentation in some patients without previous diagnosis of prostatic adenocarcinoma [4]. In many patients, the prostatic carcinoma is either impalpable or encountered incidentally after transurethral resection for benign prostatic hyperplasia [5,6], in which situation the patients may potentially have metastases without knowing the presence of prostatic primary. Therefore, in surgical pathology practice, a metastatic prostatic adenocarcinoma is always included in the differential diagnosis when encountering a male patient with metastatic adenocarcinoma of unknown origin.

Immunohistochemical staining with prostate specific antigen (PSA) is widely used to aid in the diagnosis of metastatic prostatic carcinoma. However, PSA may not be expressed in all cases of prostatic adenocarcinoma [7], especially in some poorly differentiated prostatic carcinomas or metastatic carcinoma [8-11]. Prostatic acid phosphatase (PAP) did not show better sensitivity than PSA in this regard [12,13]. In addition, immunoreactivity of PSA has been found in some non-prostatic tissues [14-17].

P501s (prostein) is a prostate-specific marker that is expressed in the cytoplasm of benign and malignant prostatic glandular cells [18-21]. Prostein is a 553 amino acid protein which contains 11 potential transmembrane spanning domains [21]. It has not been detected in any other normal or malignant tissues [19,21]. There is no correlation between prostein gene expression and the prostatic carcinoma Gleason score [21]. Further, no gross difference in the level of protein expression between primary and metastatic prostatic carcinomas is observed [21]. These features of prostein may make it a good immunohistochemical marker for identification of metastatic prostate adenocarcinoma. The purpose of this study was to further characterize the p501s staining features, especially in metastatic prostatic adenocarcinoma, and to compare its expression with PSA for the diagnosis of metastatic prostate carcinoma.

## Methods

### Construction of Tissue Microarray Blocks

Tissue microarray (TMA) blocks were created from specimens retrieved from the surgical pathology archives of the University of Pittsburgh Medical Center. There were 24 cases of normal donor prostates (NDP), 135 of acinar type of prostatic adenocarcinoma (PCA), 36 of non-neoplastic prostatic tissues adjacent to malignant glands (NNT), 35 of benign prostatic hyperplasia (BPH), 35 of high-grade prostatic intraepithelial neoplasia (PIN), 36 of metastatic adenocarcinoma to lymph nodes (MLN), and 24 of metastatic carcinoma to other sites (MC). In addition, samples of benign testis, colon, adrenal and kidney were also included (n = 6 each). TMA blacks were constructed from multiple paraffin-embedded tissue blocks by sampling a specific region from each block in the form of a cylindrical core and then assembling these tissue cores from the different donor blocks into a new composite paraffin block, as previously described [22]. To address the tissue heterogeneity, four core samples were taken from each paraffin-embedded tissue specimen.

### Immunohistochemistry

Immunohistochemical stains were performed on five-micron sections of TMA blocks. The sections of all the groups were deparaffinized and rehydrated through a graded series of ethanol. Heat induced epitope retrieval was performed using decloaker, followed by rinsing in TBS buffer for 5 minutes. Slides were then loaded on Dako Autostainer. The primary anti-p501s (working dilution 1:400) was a monoclonal mouse antibody (Clone 10E3, Code M3615) for DakoCytomation (Dako North America, Inc., Carpinteria, CA, USA). The immunolabeling procedures were carried out according to manufacturers' instruction using Dako Envision Labelled Polymer-HRP anti-mouse/anti rabbit (Dako, Glostrup, Denmark). Slides were then counterstained in hematoxylin and coverslipped.

The two groups of metastatic lesions were also subjected to immunohistochemical stains as described above with antibodies to PSA, which was a pre-diluted ready-to-use rabbit polyclonal antibody (Code N1517) for DakoCytomation (Dako North America, Inc., Carpinteria, CA, USA). A prostate optimization MRA block was used as positive control for each antibody.

### Interpretation of immunohistochemical stains

In scoring the expression of p501s and PSA, both the extent and intensity of immonopositivity were considered, using a method similar to Zhao, et al [23]. The intensity of positivity was scored from 0 to 3 as follows: 0 as non-stained, 1 as weak, 2 as moderate, and 3 as strong as positive control. The percentage of positively stained cells for each staining-intensity was estimated. The final composite score was determined after multiplying the intensity of positivity and percentage of positivity in the respective lesions. For example, if 50% of tumor cells are scored 1, 25% scored 2, and the remaining 25% scored 3, the composite staining score of this case is [50% × 1] + [25% × 2] + [25% × 3] = 1.75. Two board certified pathologists evaluated the specimens of this study. The data was presented as mean ± standard errors (SE).

## Results

**Figure 1:**
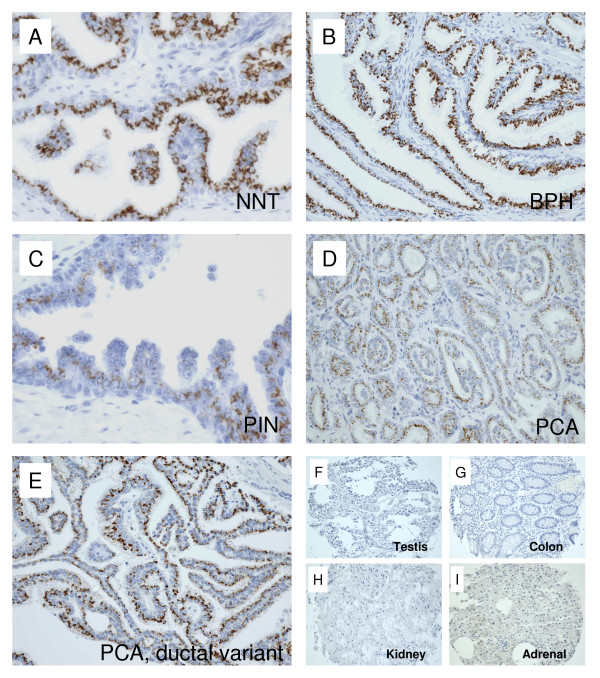
**Immunohistochemical expression of p501s on benign and malignant epithelium of prostate and non-prostatic tissues**. A. Non-neoplastic prostatic tissue adjacent to malignant glands (NNT). B. Benign prostatic hyperplasia. C. High-grade prostatic intraepithelial neoplasia (PIN). D. Prostatic adenocarcinoma (PCA).

**Figure 2:**
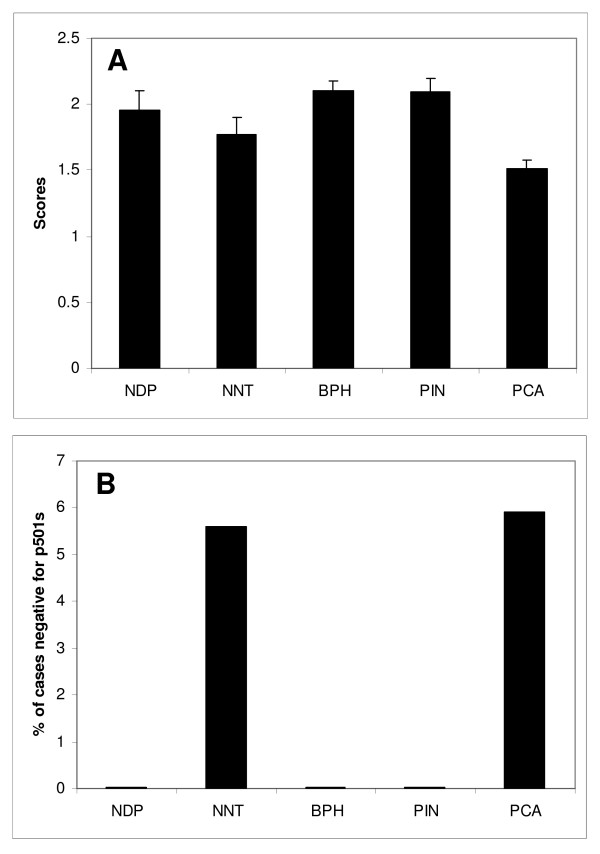
**Scores of p501s expression and percentage of cases negative for p501s**. A. Mean scores of p501s immunohistochemical expression in benign and malignant prostatic epithelium. NDP: normal donor prostate. NNT: non-neoplastic prostatic tissues adjacent to malignant glands of prostatic adenocarcinoma. BPH: benign prostatic hyperplasia. PIN: high-grade prostatic intraepithelial neoplasia. PCA: prostatic adenocarcinoma. B. Percentage of cases of each group that were negative for p501s staining.

Immunohistochemical analysis using anti-p501s antibodies was performed in benign and malignant epithelial cells of prostate. A granular staining at the apical aspect of cytoplasm, predominantly adjacent to the nuclei, was observed (Figure 1A–E, ), corresponding to the location of Golgi complex[21]. In weakly stained cases, the granules can be relatively faint and punctuate, but still be visible in the apical region of the cells using higher magnifications. The staining scores for NDP, BPH and PIN were 1.95 ± 0.15, 2.1 ± 0.8 and 2.09 ± 0.1, respectively (Figure 2A). None of the cases from the three groups were negative for p501s. The non-neoplastic tissues adjacent to malignant glands (NNT) received a score of 1.77 ± 0.13 (Figure 2A), with 5.6% of the cases showing loss of p501s expression (Figure 2B). The mean score for PCA cases was 1.52 ± 0.06, which was not significantly different from that of the above non-neoplastic groups. In the PCA group, 5.9% of cases were negative for p501s (Figure 2B). The prostatic ductal carcinoma demonstrated a similar p501s-staining pattern (Figure 1E). No staining was seen in samples of testis, colon, and kidney (Figures 1F–1H). Some adrenal tissues revealed a very weak, smudge, and non-granular cytoplasmic staining, which was considered non-specific (Figure 1I).

**Figure 3:**
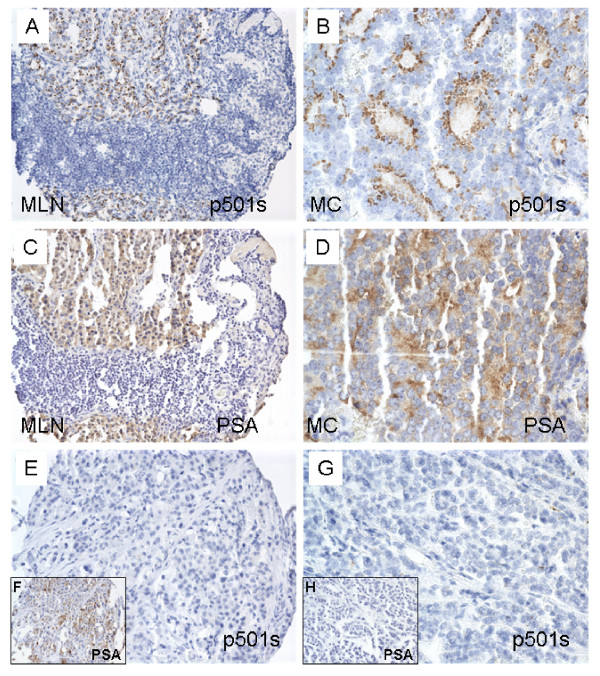
**Immunohistochemical expression of p501s and PSA on metastatic prostatic carcinoma**. A and C are sections of metastatic prostatic carcinoma to lymph node (MLN) from the same TMA block. B and D are sections of metastatic prostatic carcinoma to other sites (MC) from the same TMA block. Panels E and F are sections from the same MC TMA block; panels G and H are from another MC TMA block.

**Figure 4:**
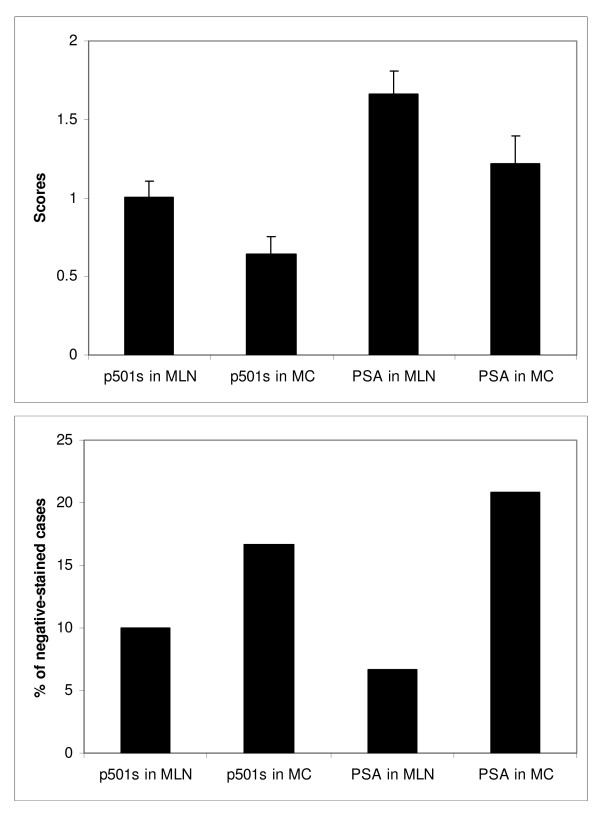
**Scores of p501s and PSA expression in metastatic prostatic lesions (A) and percentage of cases negative for p501s and PSA stains (B)**. MLN: metastatic prostatic carcinoma to lymph nodes. MC: metastatic prostatic carcinoma to other sites.

Metastatic prostatic carcinomas also demonstrate a granular apical staining pattern with p501s antibody (Figures 3A and 3B). The mean scores of p501s staining in the MLN and MC groups were 1.0 ± 0.1 and 0.64 ± 0.11 (Figure 4A), respectively, which were statistically lower than those of the above benign and malignant epithelium of prostate per se (p < 0.05) (see Table 1 for the summary of p501s staining scores). Negative p501s staining was seen in 3 of 30 MLN cases (10%) and in 4 of 24 MC cases (16.7%) (Figure 4B). P501s negativity was found in 13% of all metastatic lesions (combined MLN and MC).

**Table 1:** Summary of p501s staining scores on benign and malignant prostatic epithelium

	LESIONS	MEAN SCORE ± SE
Benign prostatic epithelium	Normal donor prostates (NDP)	1.95 ± 0.15
	Non-neoplastic prostatic tissues adjacent to malignant glands (NNT)	1.77 ± 0.13
	Benign prostatic hyperplasia (BPH)	2.10 ± 0.08
Malignant prostatic epithelium	High-grade prostatic intraepithelial neoplasia (HGPIN)	2.09 ± 0.10
	prostatic adenocarcinoma (PCA)	1.52 ± 0.06
Metastatic prostatic carcinoma	Metastatic carcinoma to lymph nodes (MLN)	1.0 ± 0.10
	Metastatic carcinoma to other sites (MC)	0.64 ± 0.11

Loss of PSA expression was observed in 2 of 30 MLN cases (6.7%) and in 5 of 24 MC cases (20.8%) (Figure 4A). In the PSA immunoreactive metastatic lesions, a denser diffuse cytoplasmic PSA staining was seen (Figures 3B and 3D). As a result of the diffusing staining pattern of PSA, the mean scores of PSA from the metastatic lesions were slightly higher than those with p501s antibody (1.66 ± 0.15 for MLN and 1.22 ± 0.18 for MC, respectively) (Figure 4B). However, it was clear that different staining patterns were present when comparing the two antibodies.

Some of the MC cases were negative for p501s, but positive for PSA (Figures 3E and 3F). In contrast, some MC cases were negative for PSA, but immunoreactive for p501s (Figures 3G and 3H). In high-grade metastatic carcinoma, the p501s stain may be weak and punctuated (Figure 3F). None of MLN cases showed loss of both p501s and PSA. Simultaneous negative stains for both p501s and PSA were seen in only 2 MC cases (5.6%), which comprised 3.3% of all metastatic lesions.

## Discussion

P501s (prostein) is an organ specific marker for benign and malignant prostatic epithelial cells. The previous and current studies did not find the p501s expression in any other organ systems at both mRNA and protein levels [19,21]. This makes p501s a great candidate for the development of new diagnostic as well as prostein-specific antibody and T-cell based therapeutic strategies against prostatic carcinoma [20]. The staining pattern of p501s was granular discoloration at the apical aspect of cytoplasm of epithelial cells, which was thought to correspond to the Golgi complex [21]. Therefore, in addition to its organ specificity, the characteristic stain pattern of prostein provides an additional valuable immunomarker for detection of metastatic carcinoma of prostatic primary. However, in some cases, the p501s-stained granules can be relatively faint and punctuate, we suggest that high-power examination is necessary to rule out focal staining.

In the metastatic prostatic lesions, the staining intensity of p501s was reduced. Likewise, the loss of p501s expression was seen in a small proportion of the metastatic lesions. This phenomena was also observed in previous studies with PSA stains [8-11], but argued against the previous statements made by Kalos and coworkers in this regard [21]. It was found that PSA immunoreactivity declined from benign epithelium to PIN and prostatic adenocarcinoma, suggesting that PSA is regulated differentially and decreased in expression with malignant transformation [10]. The mechanisms responsible for the diminished expression of p501s in metastatic prostatic carcinomas are unknown, but could be similar to those for PSA. Interestingly, there was a trend of decreased p501s expression in the groups of primary prostatic carcinoma (PCA) and non-neoplastic prostatic tissue adjacent to malignant glands (NNT) (Figure 2), although no statistical differences were identified compared with groups of NDP, BPH and PIN. Since no Gleason score was given to the metastatic lesions, it was possible that metastatic carcinomas represent higher-grade cancers. As a result of loss of p501s expression in some metastatic lesions, use of p501s alone did not offer a significant advantage in identifying the metastatic tumors when comparing with the use of PSA alone. However, simultaneous stains with P501S and PSA largely improved the detection rate and identify a significant majority of the metastases. In fact, only 2 cases (3%) were concurrently negative for both p501s and PSA. This increases the diagnostic utility of using these immunostains when used together in the assessment of an unknown primary, possibly of a prostatic origin. Recently, Netto and his coworkers also concluded that the combination of PSA and p501s is better than either alone in confirming prostate origin in work-up of metastatic or locally advanced lesions [24].

## Conclusion

P501s is an organ specific marker for benign and malignant prostatic epithelial cells. Its characteristic cytoplasmic stain pattern provides an additional valuable immunomarker for detection of metastatic prostatic malignancy, even though the intensity of its expression is reduced, as in the case with PSA. Simultaneous stains with P501S and PSA will greatly improve the detection rate and identify a significant majority of the metastases. Our study highlights the importance of including P501s in a diagnostic immunostaining panel when the differential diagnosis includes a poorly differentiated carcinoma of an unknown primary and may play in pivotal role in the diagnosis of such lesions.

## Competing interests

The author(s) declare that they have no competing interests.

## Authors' contributions

MY participated in the design of the study and the histopathological evaluation, performed the literature review, acquired the photomicrographs, and drafted the manuscript. AVP conceived and designed the study, gave the final histopathological diagnosis, revised the manuscript for important intellectual content, and made the decision to submit the manuscript for publication. RD also gave the final histopathological diagnosis, participated in the coordination of the study, and revised the manuscript for important intellectual content. All authors read and approved the final manuscript. The source of funding for these studies was from The Department of Pathology at The University of Pittsburgh.
